# Role of 1q21 in Multiple Myeloma: From Pathogenesis to Possible Therapeutic Targets

**DOI:** 10.3390/cells10061360

**Published:** 2021-06-01

**Authors:** Jessica Burroughs Garcìa, Rosa Alba Eufemiese, Paola Storti, Gabriella Sammarelli, Luisa Craviotto, Giannalisa Todaro, Denise Toscani, Valentina Marchica, Nicola Giuliani

**Affiliations:** 1 Department of Medicine and Surgery, University of Parma, 43126 Parma, Italy; jessica.burroughsgarcia@unipr.it (J.B.G.); eufemieserosalba@gmail.com (R.A.E.); paola.storti@unipr.it (P.S.); luisa.craviotto@unipr.it (L.C.); denise.toscani@unipr.it (D.T.); valentina.marchica@unipr.it (V.M.); 2 Department of Medical-Veterinary Science, University of Parma, 43121 Parma, Italy; 3 Hematology, Azienda Ospedaliero-Universitaria di Parma, 43126 Parma, Italy; gsammarelli@ao.pr.it (G.S.); giannalisat@gmail.com (G.T.)

**Keywords:** multiple myeloma, 1q21, chromosome aberrations, amplification, *IL6R*, *ILF2*, *MCL-1*, *CKS1B*, *BCL9*

## Abstract

Multiple myeloma (MM) is characterized by an accumulation of malignant plasma cells (PCs) in the bone marrow (BM). The amplification of 1q21 is one of the most common cytogenetic abnormalities occurring in around 40% of *de novo* patients and 70% of relapsed/refractory MM. Patients with this unfavorable cytogenetic abnormality are considered to be high risk with a poor response to standard therapies. The gene(s) driving amplification of the 1q21 amplicon has not been fully studied. A number of clear candidates are under investigation, and some of them (*IL6R*, *ILF2*, *MCL-1*, *CKS1B* and *BCL9*) have been recently proposed to be potential drivers of this region. However, much remains to be learned about the biology of the genes driving the disease progression in MM patients with 1q21 amp. Understanding the mechanisms of these genes is important for the development of effective targeted therapeutic approaches to treat these patients for whom effective therapies are currently lacking. In this paper, we review the current knowledge about the pathological features, the mechanism of 1q21 amplification, and the signal pathway of the most relevant candidate genes that have been suggested as possible therapeutic targets for the 1q21 amplicon.

## 1. Introduction

Multiple myeloma (MM) is characterized by an abnormal proliferation of plasma cells (PCs) in the bone marrow (BM). Despite the significant improvement in overall survival (OS), most MM patients eventually relapse and develop refractory disease [1]. MM is a genetically complex disease characterized by a distinct clinical heterogeneity in the response rate and survival outcomes. Nevertheless, the progression of the disease demonstrates a wide range of heterogeneity, with the OS time ranging from less than 12 months to more than 10 years [2,3]. The heterogeneity in MM can be explained either by the intrinsic genetic heterogeneity of PCs or at least in part by the BM microenvironment, which generates a high-risk environment that facilitates cancer cell survival [4,5,6].

**Figure 1:**
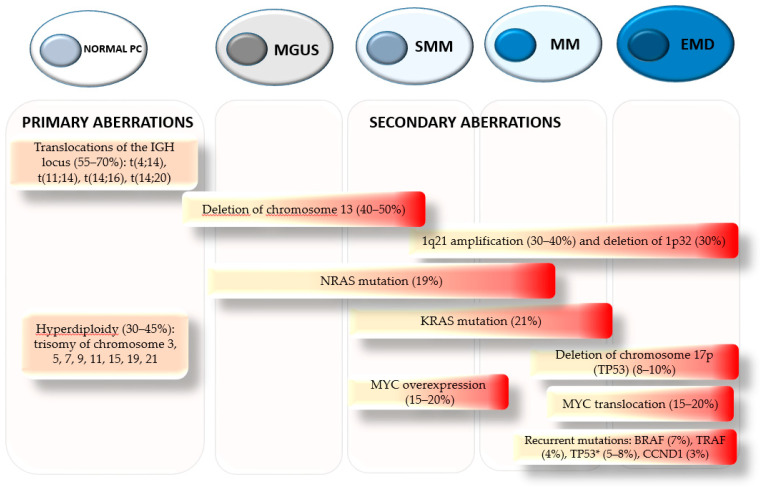
Genetic events from initiation to progression in multiple myeloma pathogenesis. Chromosomal aberrations involving immunoglobulin heavy chain (*IGH*) at the 14q32 region and the hyperdiploid are considered primary translocations as they are mutually exclusive and present in asymptomatic stages. Secondary aberrations follow primary events contributing to tumor progression and relapse. Secondary events cooperate with primary events to produce the malignant PC phenotype. The progression from MGUS-SMM to MM is associated with RAS mutation, MYC overexpression, and amplification of 1q21. Incidence of MYC translocation, deletion of 17p, and recurrent mutations increased as disease progressed. The initiation of aberrant clones at the onset of the disease is indicated in yellow. Red indicates the accumulation of malignant clones during disease progression. Percentage is indicated at new diagnosis. Asterisk indicates P53 mutation.

The presence or absence of recurrent chromosomal abnormalities is considered one of the major prognostic landmarks for MM patients. Primary immunoglobulin translocations (t) involving immunoglobulin heavy chain (*IGH*) at the 14q32 region including t(4;14), t(14;16), t(14;20), and the hyperdiploid including trisomies of the odd-numbered chromosomes, occur as initiating events during tumor pathogenesis (Figure 1). Secondary abnormalities occur later during disease progression. The most frequent secondary abnormalities can be classified as translocations, mutations, deletions, and amplifications (Figure 1). *MYC* translocation is found in about 15% of MM at diagnosis and 50% of more advanced stages [7]. Approximately one-third of *MYC* translocations involve rearrangements with the *IGH* locus [8,9]. In addition, copy number change at the *MYC* locus is found in around 20% of NDMM patients [9]. The overexpression of *MYC* is typically associated with a poor prognosis in patients with MM. The *RAS* gene family (*NRAS* and *KRAS*) is one of the most frequently mutated groups of genes in MM [10]. The prevalence of both *NRAS* and *KRAS* mutations in MM patients is between 20 and 35%. It has been suggested that *KRAS* plays a more influential role in the pathogenesis of MM. *RAS* mutations are usually associated with less favorable disease outcomes, greater tumor burden, and shortened survival time [10]. The tumor suppressor gene *TP53* is located on chromosome 17 (17p). Deletions of the 17p region can lead to monoallelic inactivation of *TP53* [11]. Recently, bi-allelic inactivation of *TP53* has been associated with high risk, poor OS, and resistance to conventional MM treatments [12,13]. In MM, *TP53* mutations are uncommon at diagnosis and represent late events in disease progression, suggested to play an essential role in MM pathogenesis [13,14]. In a multivariate analysis, D’Agostino et al. showed that mutation of *P53* was correlated with early relapse in MM patients. In addition, the study showed that patients with both, deletion of the 17p and *P53* mutations, have a shorter OS compared to patients with only one chromosomal aberration [15]. An increased risk of acquired *P53* mutation was also observed in patients carrying a deletion of 17p [15]. Along with *TP53* mutation, deletion of chromosome 13 has also been associated with adverse disease outcome [16].

Amplification of chromosome 1q (1q21 amp) is one of the most common secondary cytogenetic abnormalities in patients with MM. 1q21 amp is often associated with poor prognosis, drug resistance, and disease progression [17]. The 1q21 region is known to contain several oncogenes and genes that may show simultaneous amplification and/or deregulated expression. To date, the relevant gene(s) driving the high-risk disease progression associated with 1q21 amp has not been fully identified, suggesting that more than one candidate gene may be responsible for the poor outcome in this group of patients. In this review, we provide an overview of the pathological features, prognostic values, and the signal pathway of several target genes located on the 1q21 region. This review can shed light on alternative therapeutic approaches to treat MM patients carrying 1q21 amp that usually do not benefit from currently available treatments.

## 2. Pathogenesis and Clinical Implications of 1q21 Amplification

The 1q21 region is gained (three copies) or amplified (≥ four copies) in about 40% of *de novo* cases and 70% of the relapsed–refractory (RR) MM patients (Figure 1) [18,19]. Patients with this unfavorable cytogenetic abnormality are considered to be high risk with a poor response to standard therapies [17,20]. The amplification of 1q21 commonly occurs as arm-level aberrations or focal amplifications, and both cases are the result of the genomic instability from the 1q12 region [21,22]. In several studies, Sawyer et al. underlined the mechanism for the amplification of 1q21 in MM [21,22,23,24]. He suggested that the amplification of the 1q region occurs when duplication of the 1q12 pericentriomeric region translocates, leading to jumping segmental duplications of the chromosome 1q band, resulting from de-condensation of the pericentromeric heterochromatin region [21,22,23,24]. A second type of 1q arm-level copy number alteration commonly seen is the formation of isochromosomes, which results in the duplication of one arm and the loss of the other, resulting in an amplification of the 1q21 region [23]. The 1q21 amplified region can be detected by fluorescent in situ hybridization (FISH) [18]. This method has become a standard clinical feature for the identification of patients with 1q21 amp [18].

Studies have shown that gain and/or amplification of 1q21 can be found at any stages of MM disease, from monoclonal gammopathy of uncertain significance (MGUS) (15–20%), to smoldering (SSM) (30%), while in highly proliferative disease such as MM and extramedullary disease (EMD) the accumulation of additional copies of 1q21 results in as much as 70% of patients having more than three copies (Figure 1) [18,25,26]. In fact, the heterogeneous genetic background of patients with 1q21 amp has a profound impact on survival when compared to those with 1q21 gain. A study by Hanamura et al. showed that patients with amplification of 1q21 at diagnosis tended to have a more aggressive clinical course than those with 1q21 gain [18]. The OS was similar in both groups; however, RRMM patients carrying 1q21 amp showed a shorter PFS and OS survival due to the more aggressive tumor behavior when compared with those with 1q21 gain [18].

Immunomodulatory drugs IMMDs and proteasome inhibitors (PI) have become an essential part of the MM treatment regimen due to their well-established clinical efficacy. Both drugs have been successfully used to treat MM high-risk patients, in particular patients with t(4:14) and del(17p). In a randomized study, Cavo et al. showed that patients with t(4:14) and/or del(17p) who received a combination of PI + IMMDs + dexamethasone (VDT) after autologous stem cell transplantation (ASCT) have a longer PFS and OS than those patients who only received IMMDs + dexamethasone (TD) as a base treatment [27]. A study by Shaughnessy et al. demonstrated that patients carrying 1q21 amp treated with bortezomib showed an upregulation of proteasomes genes including *PSMD4*, a gene commonly upregulated and associated with adverse disease outcome in patients with 1q21 amp [28]. The results from this study suggest that additional copies of 1q21 may mediate the resistance to bortezomib in these patients, whom often have an early progression and a shorter OS [28]. A more recent retrospective study has shown that patients with 1q21 gain have a better response to VDT induction therapy and accomplish a very good response (VGR) and/or complete response (CR) after ASCT when compared to patients without 1q21 gain [29]. In addition to bortezomib, it has been proposed that carfilzomib, a novel second-generation PI, may be capable to overcome bortezomib resistance in high-risk MM patients [30]. Early-phase clinical studies have demonstrated that NDMM high-risk patients treated with carfilzomib-based induction achieved impressively high rates of MRD negativity and longer PFS. However, the results regarding the outcome of patients with 1q21 amp have not been published yet [30]. On the other hand, encouraging results regarding patients with 1q21 gain have come from the FORTE trial [30]. The results from this study showed that NDMM patients who received carfilzomib in combination with cyclophosphamide and dexamethasone (KRd) prior to and after ASCT have a longer PFS while patients with 1q21 amplification showed a very poor response to the treatment [30].

The coexistence of 1q21 amp with other cytogenetic aberrations has a negative impact on MM outcome. Recently, Abdallah et al. evaluated the impact of 1q21 amp on clinical characteristics and OS in more than 1300 NDMM patients. The results from a multivariate analysis show that 1q21 amp was associated with a decreased OS when other cytogenetic abnormalities, such as the loss of the short arm of chromosome 17 or translocations t(4;14), t(14;16), and t(16;20), were included [31]. This prognostic value was retained when advanced ISS stage and older age was considered [31]. In another study, Walker et al. characterized a double-hit subgroup in NDMM patients. This cohort of patients was described to have either the bi-allelic inactivation of TP53 or ISS stage III with more than four copies of 1q21. The double-hit was rare, present in only 6.1% of the patients in the study [32]. In addition, a study performed by Jin et al. found that patients with concomitant 1q21 amp and *MYC* rearrangement presented a higher frequency of EMD and hypercalcemia than patients with 1q21 amp or *MYC* rearrangement alone [33]. The rate of CR and VGR was relatively lower in patients with a combination of 1q21 amp and *MYC* rearrangement when compared to patients carrying only one aberration [33].

## 3. 1q21 Amplification and Potential Druggable Targets Genes

**Figure 2:**
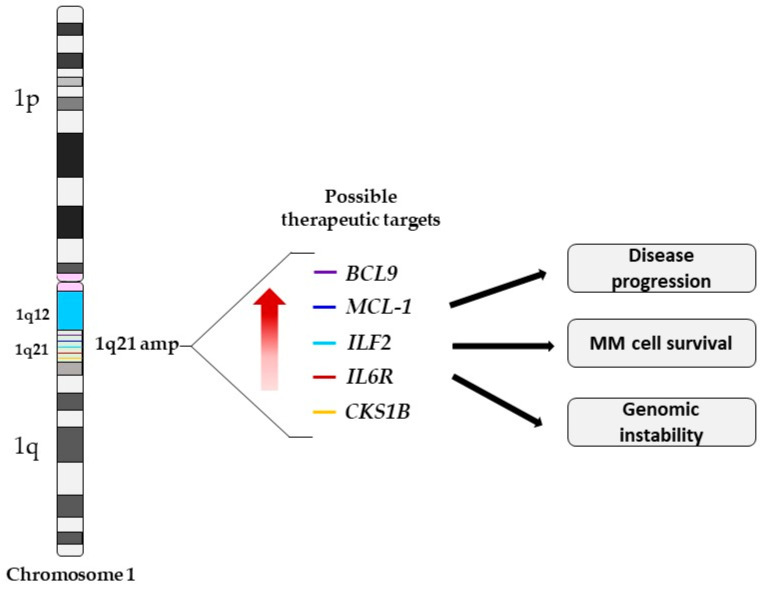
Schematic representation of chromosome 1 indicating the location of the possible genetic driver genes in this region, denoted by colored lines. The amplification of the 1q21 region results in the simultaneous overexpression of several genes leading to disease progression, MM cell survival, and an increase in genomic instability. *BCL9*, B cell lymphoma 9; *MCL-1,* myeloid cell leukemia-1; *ILF2*, interleukin enhancer binding factor 2; *IL6R*, interleukin-6 receptor; and *CKS1B*, CDC28 protein kinase regulatory subunit 1B.

Amplification of 1q21 is an important prognostic marker in MM. This region contains a large number of genes including *IL6R*, *MCL-1*, *ILF2*, *BCL9* and *CKS1B* that have been reported to drive disease aggressiveness in 1q21 amp cases (Table 1). The amplification of the 1q21 region often results in the simultaneous upregulation and/or deregulated expression of these genes, which frequently correlates with poor prognosis and drug resistance (Figure 2) [28,34,35,36,37]. The exact gene(s) driving the amplification of 1q21 have not been fully characterized. Several candidate genes have been proposed as potential 1q21 targets. In this part of the review, we provide a summary of the mechanisms of the most relevant MM targetable genes located on the 1q21 region.

### 3.1. MCL-1

The myeloid cell leukemia-1 (*MCL-1*) belongs to the BCL-2 family [46]. Both *MCL-1* and *BCL-2* have functional similarities and share the ability to promote cell survival [47]. The RNA expression of both genes is independently regulated and the tissue distribution shows significant differences, suggesting that both proteins may have different roles in controlling apoptotic pathways [48]. High expression levels of *MCL-1* are commonly seen in hematological cancers [49]. *MCL-1* is known to act as a gene inducing tumor progression [38]. The *MCL-1* transcriptional regulation is cell type-dependent, modulated by several extracellular stimuli such as growth factors, endothelial growth factors, interferons, and cytokines such as interleukin 6 (IL6), which plays a critical role in MM progression [38]. In addition, several cytoplasmic pathways (e.g., PI3K/Akt, JAK/STAT, and MEK/ERK) have been shown to be activated by extracellular stimuli affecting the downstream expression of MCL-1 [38]. The expression of *MCL-1* can be regulated in a paracrine fashion by IL6. *IL6* binds its receptor and activates Janus family kinase-2 (JAK2), thereby inducing the signal transducer activator of the transcription 3 (STAT3) homodimerization [50]. Once homodimerized, STAT3 translocates to the nucleus, increasing *MCL-1* transcription [50]. *MCL-1* expression can also be independently regulated from other survival signals from the BM microenvironment, such as interferon α (IFNα) which induces MCL-1 in a STAT3-dependent manner [50]. In many cancers, overexpression of *MCL-1* contributes to the development of malignancies and it is often associated with resistance to conventional therapies. The role of *MCL-1* in MM has been evaluated in several studies looking at the effect of *MCL-1* expression levels on cell proliferation [51]. Studies have demonstrated that expression of *MCL-1* is important for B lymphocyte development and PC survival [52]. Wuilleme-Toumi et al. evaluated the expression of *MCL-1* in BM PCs from MM patients at diagnosis and after relapse [35]. They found that overexpression of *MCL-1* leads to apoptosis resistance associated with disease progression and shorter OS [35]. *MCL-1* is located in the 1q21 amplicon. Notably, patients carrying 1q21 amp have an increase in *MCL-1* expression which correlates with a poor OS [53]. *MCL-1* overexpression is frequently observed and appears to be a key factor in resistance. Therefore, targeting *MCL-1* may be an effective approach to treat this group of MM patients that do not benefit from current therapies. The BCL2 inhibitor venetoclax has been approved to treat MM patients in 2016 [54,55,56]. Studies showed that therapy combining bortezomib and dexamethasone obtained a better response only in RRMM patients with high expression levels of *BCL2* [55].

Targeting *MCL-1* itself will be a more promising therapeutic approach for MM patients carrying 1q21 amp. MCL-1 inhibitor S63845 has shown promising results for MM patients with 1q21 amp [57]. A study conducted by Slomp et al. showed that primary BM MM cells from patients carrying 1q21 amp were significantly more sensitive to treatment with MCL-1 inhibitor S63845 than MM cells from patients without this chromosomal aberration, suggesting that S63845 may be more effective in MM patients with 1q21 amp [53]. The MCL-1 inhibitor S64315/MIK665 is currently being tested for MM in phase I trial. Overall, the identification of *MCL-1* inhibitors will offer a great advance for the generation of effective treatment that could benefit MM patients with 1q21 amp.

### 3.2. CKS1B

The CDC28 protein kinase regulatory subunit 1B (*CKS1B*) belongs to the cyclin kinase subunit 1 protein family. It is known to play a pivotal role in cell proliferation [39]. CKS1B was identified as a protein component of the SCF^SKP2-CKS1^ ubiquitin ligase complex [39,58]. In this complex, CKS1B enhances the interaction between p27^Kip1^ (a CDK inhibitor) and SKP2, leading to cell proliferation [59]. The amplification of *CKS1B* results in the regulation of the p27^Kip1^ proteasome degradation in the cell cycle, and increased mitotic activity [40]. *CKS1B* is one of the most commonly overexpressed genes in MM located at the 1q21.3 region [39]. Studies by Shaughnessy et al. showed that upregulation of *CKS1B* is positively associated with an increased copy number in the 1q21 region [17]. In MM patients overexpression of *CKS1B* results in a shorter PFS and decreased OS [17,60]. In addition, CKS1B expression is strongly correlated with increased copy number in bortezomib RRMM patients [61]. Evidence suggests that MM cells with more than four copies of 1q21 are potentially associated with a more adverse phenotype and drug resistance than those with three copies. Studies by Shi et al. demonstrated that *CKS1B* mediates MM cell proliferation and chemoresistance through the activation of STAT3 and MEK/ERK/BCL2 signaling pathways [62]. The results from this study show that upregulation of CKS1B triggers STAT3 and MEK/ERK signaling pathways, showing that these pathways are the downstream signaling pathways of CKS1B [62]. *CKS1B* overexpression is frequently observed in patients with 1q21 amp conferring resistance to MM chemotherapeutic agents. Considerable evidence indicates that targeting *CKS1B* is beneficial for the treatment of patients with 1q21 amp. Recent studies have provided preliminary insights about the different ways to target this novel gene. Huang et al. investigated the effect of MLN4924, a novel ubiquitin-like inhibitor involved in the process of ubiquitin-like modification [63]. MLN4924 selectively inhibits NAE and prevents the formation of the SCF^Skp2^ complex. The results from this study show MM cells overexpressing *CKS1B* are more sensitive to MLN4924 treatment. Treatment of *CSK1B* overexpressing MM cells with MLN4924 results in a decrease in cell proliferation and the induction of cellular senescence [63].

In another study, Malek et al. identified DT204, a novel compound able to inhibit degradation of p27 mediated by SCF^Skp2^, preventing Skp2 incorporation into the SCF^Skp2^ complex [64]. DT204 has been shown to reduce myeloma viability by inducing cell cycle arrest and enhancing the anti-myeloma effect of BTZ in MM cell lines and patient samples [64]. Recently, using the COMPARE algorithm to identify the correlation between *CKS1B* gene expression and drug activity, Tian et al. identified 9-dimethyl amino-ethoxy ellipticine (EPED3), a highly stable compound derivative of the plant alkaloid ellipticine [65]. Studies using MM cells showed that treatment with EPED3 induced cell death. Similar results were obtained using cells with acquired resistance to anti-myeloma drugs [65]. Therapeutic strategies targeting *CKS1B* may hold promise for the treatment of MM patients with 1q21 amp. Targeting *CKS1B* will allow the design of better therapeutic strategies to overcome adverse prognosis and drug resistance observed in patients with 1q21 amplification. However, further clarification on the mechanism underlying *CKS1B* function and its regulation will provide additional insights on the best way to target this gene.

### 3.3. ILF2

The interleukin enhancer binding factor 2 (*ILF2*) encodes nuclear factor (NF) 45, the regulatory subunit of NF90/NF110 complexes that have been reported to play essential roles in mRNA stability, post-transcriptional regulation, translation, and in mitotic control [42,43]. The potential role of *ILF2* as a therapeutic target in 1q21 region has been demonstrated by Marchesini et al. [36]. The authors showed that *ILF2* is a key modulator of the homologous recombination (HR) DNA repair pathway in myeloma cells [36]. Overexpression of *ILF2* promotes tolerance of genomic instability, thereby enhancing MM cell survival and drug resistance [32].

At a mechanistic level, Marchesini et al. demonstrated that increased *ILF2* expression drives resistance to genotoxic stress agents by regulating the nuclear localization of the Y box-binding protein 1 (YB-1) and its interplay with the splicing factor U2AF65, promoting mRNA splicing of transcripts involved in HD DNA repair [36]. In line with these findings, clinical observations from the same research group also demonstrated that nuclear expression of ILF2 strongly correlates with the expression of YB-1 in MM patients with 1q21 amp [36]. Notably, the work by Marchesini et al. supports the possible clinical use of *ILF2* as a potential therapeutic target for 1q21 [36]. Blocking expression of *ILF2* in MM patients with 1q21 amp could enhance the efficacy and overcome resistance to DNA-damaging drugs such as melphalan and cyclophosphamide [36].

### 3.4. IL6R

The interleukin-6 receptor (*IL6R*) is a critical gene located within the amplified 1q21 region [66]. *IL6R* has been documented to be a predictor of poor outcome in MM patients [66,67,68,69]. IL6R is part of the ligand-binding domain of IL6, a pleiotropic cytokine required for the terminal differentiation of B cells [70]. IL6 has been shown to be a potent driver for MM disease progression [71]. Elevated levels of IL6 in MM patients are often associated with prevention of drug-induced-apoptosis and disease progression [66,71,72,73]. IL6R is a complex associated with gp130. The association of this complex activates tyrosine phosphorylation of JAK and others downstream pathways such as STAT3 and the RAS/mitogen-activated protein kinase (MAPK) [71,74,75,76,77]. Once phosphorylated by JAK, STAT3 dimerizes and translocates to the nucleus, leading to the transcription of various pro-survival and anti-apoptotic genes, such as *MCL-1* and *BCL-2* [78,79].

STAT3 was found to be constitutively active in primary MM tumors and cell lines [80]. Activation of IL6/STAT3 pathway was correlated with drug resistance and adverse outcome [80,81,82]. Studies using MM cells have revealed that the STAT3 pathway is constitutively active in around 48% of patients. The over-activation of STAT3 resulted in poor survival and drug resistance [82]. In a study using CRISPR-associated protein-9 knockout screening in MM cell lines, Ogiya et al. identified that the JAK–STAT3 pathway mediates CD38 downregulation [83]. Pharmacological inhibition of the JAK–STAT3 pathway results in STAT3 phosphorylation in MM cell lines, an upregulation of CD38 expression in primary MM patients, and an increase in daratumumab antibody-mediated cellular cytotoxicity against MM cell lines [83]. Several studies have demonstrated that inhibition of the IL6R/STAT3 pathway with anti-IL6, IL6R antagonists, or JAK-inhibitors induced apoptosis of human myeloma cell lines in vitro and enhanced the antitumor activity of other anti-myeloma drugs in vivo, attenuating chemoresistance [84,85,86,87]. However, the outcome in clinical trials using anti IL6 antibodies was less significant, probably due to the lack of understanding of the IL6/STAT3 pathway [3].

Recently, using mRNA from patients’ BM PCs, it was demonstrated that the expression level of *IL6R* was associated with the 1q21 copy number [88,89]. Teoh et al. reported that amplification of the 1q21 region results in an increased expression of *IL6R* and *ADAR1* (an RNA-editing enzyme); both critical genes are located on the 1q21 region [34]. The concomitant gain of *IL6R* and *ADAR1* resulted in MM cell proliferation through the hyper-activation of the STAT3 pathway, conferring hypersensitivity of IL6, and as a consequence, driving the over-activation of the STAT3 pathway in cells with 1q21 amp [34]. These results explain why targeting IL6 may not be sufficient for these patients. Targeting *IL6R* along with other components of the complex, such as STAT3, might be more beneficial. However, further studies focusing on the understanding of the mechanism of action of the *IL6R, ADAR1*, and STAT3 pathways will provide better insights for the development of effective treatments for high-risk patients with 1q21 amp.

### 3.5. BCL9

The B cell lymphoma 9 (*BCL9*) gene also resides on chromosome 1q21. BCL9 is one of the nuclear Wnt pathway components and plays an important role in the transcriptional activity of β-catenin [44]. The dysregulation of the canonical Wnt/β-catenin pathway and the accumulation of nuclear β-catenin have been implicated in numerous human malignancies, including MM [45]. Gene-expression analysis revealed that *BCL9* is overexpressed in approximately 60% of late-stage MM patients and in MM cell lines [90]. Mani et al. showed that *BCL9* knockdown in MM cell lines reduced cell proliferation and colony-forming activity, probably due to the decreased cell cycle progression or increased apoptosis, whereas overexpression of *BCL9* in MM1S cells significantly increased the colony-forming ability. Therefore, studies in NOD/SCID mice transplanted with MM1S *BCL9* shRNA cells showed an increased in OS, fewer GFP tumor nodules, and smaller foci of cells infiltrating the bone marrow [45]. Takada et al. investigated the pharmacologic blockade of the β-catenin–BCL9 complex by the use of a stabilized alpha-helix of BCL9 (SAH-BCL9) [91]. He showed that SAH-BCL9 was able to target β-catenin, dissociates native BCL-9/β-catenin complexes, selectively suppresses Wnt transcription, and exhibits mechanism-based antitumor effects [91]. These data indicate that targeting *BCL9* may reduce tumor formation and resistance to therapy, resulting as a consequence of *BCL9* upregulation, emphasizing the importance of BCL9 as a target for the 1q21 region.

MicroRNAs (miRNA) are able to regulate RNA by modulating the expression of a particular gene. Several studies have identified miRNAs to play a role during MM development [92,93,94,95]. Recently, Zhao et al. identified a novel therapeutic tool to target *BCL9* using miRNA. The study showed that miR-30 was able to regulate *BCL9* expression in vitro [96]. Upregulation of miR-30s in MM cell lines leads to a decrease in cell proliferation and survival, in addition to the downregulation of *BCL9* and Wnt transcriptional activity [96]. These findings demonstrated the potential use of *BCL9* as a novel therapeutic gene to target the 1q21 region in MM.

## 4. Possible 1q21 Targets Genes Outside of Chromosome 1q

The amplification of 1q21 can drive the dysregulation of several genes outside of chromosome 1q. The dysregulation of these genes can result as a direct or indirect consequence of 1q21 amplification. Several studies have demonstrated that overexpression of *CKS1B*, which results from the amplification of the 1q21 region, leads to low expression levels and incorrect degradation of p27^Kip1^, which regulates Cdk2-E activity and the late stage of G1/S transition of the cell cycle [17,97,98]. Lower levels of p27^Kip1^ have been associated with poor prognosis in MM [99]. Protein and mRNA purified from BM PCs of 351 NDMM patients showed an inverse correlation between CKS1B and p27^Kip1^ expression levels [17]. Furthermore, knockdown of *CKS1B* in MM cell lines resulted in MM cell death and stabilization of p27^Kip1^ [97]. Correlation analysis of 94 NDMM showed that patients with lower expression of p27^Kip1^ have a shorter OS compared to patients with high-expression p27^Kip1^ protein [98]. These results show an alternative mechanism that influences MM cell growth and survival in patients with 1q21 amp. Therefore, p27^Kip1^ may be a novel therapeutic target for the 1q21 region in MM.

The anti-apoptotic proteins BCL2, MCL-1, and BCL-XL (BCL2L1) are expressed in MM cells [100]. Increased expression, particularly of MCL-1 and BCL-XL, is associated with worse patient outcome. Recently, Slomp et al. found that *MCL-1* was significantly upregulated in patients with 1q21 amplification and the *MCL-1*/*BCL2*, *MCL1*/*BCL-XL* ratios were higher in patients with 1q21 amp when compared with control patients [53]. In addition, amplified PCs from 1q21 showed an increase in sensitivity when treated with the combination of MCL-1 plus BCL2 inhibitors and an increase in cell death after treatment with MCL-1 plus BCL-XL inhibitors [53]. These results suggest that combination therapies with possible target genes outside of the 1q region could greatly benefit patients with 1q21 amplification. A more recent study identified that the expression levels of EPB41L4A (erythrocyte protein band 4.1-like 4a), a target gene for the Wnt/β-catenin pathway, were downregulated in a copy number manner in patients with 1q21 amp. Correlation analysis of over 500 MM expression profiles showed that patients with relapsed MM had lower expression of EPB41L4A than patients without recurrence disease [101]. At the moment, there are no other studies investigating the direct or indirect role of 1q21 amplification on the downregulation of EPB41L4A. More research investigating the role and the biological implications of EPB41L4A in patients with 1q21 amp will provide a better understanding of this possible target gene in MM.

## 5. Conclusions

Despite advances in MM therapy in recent years, MM remains an incurable disease with a highly heterogeneous genetic background [102,103]. Cytogenetic abnormalities are one of the most important prognostic factors for NDMM patients. Amplification of 1q21 is one of the most acquired genetic lesions associated with high risk and adverse prognostic factors. The incidence of 1q21 copy number is correlated with clinical outcome. MM patients harboring more than four copies of 1q21, in particular RRMM patients, have more adverse clinical outcomes than those with three 1q21 copies [103,104,105] The amplification of the 1q21 region can occur as isochromosomes, duplications, or jumping translocations, leading to the chromosomal instability of MM cells which may account for the genetic complexity and heterogeneity that characterize the patients carrying multiple copies of 1q21 [21,22,23]. Along with the amplification of the 1q region, increased expression and/or deregulation of several known MM related genes are shown to contribute to the disease pathogenesis. Many studies have suggested that amplification of the 1q21 region could be an independent detrimental prognostic factor in MM patients; however, amplification of 1q21 has not been uniformly accepted as part of the MM ISS high-risk stratification system. As part of the ongoing effort to further improve treatment outcomes of patients with 1q21 gain/amplification, the European Myeloma Network in collaboration with the HARMONY project is trying to revise the R-ISS risk stratification model to include 1q21 copy number alterations as part of the general prognosis of NDMM patients [106]. Therefore, it is necessary to understand the molecular mechanism of the genes upregulated at the 1q21 region in order to allow the development of individualized patient treatment. The relevant driver genes present in the 1q21 region have not been fully explored. Among these genes, candidate oncogenes such as *IL6R*, *MCL-1*, *BCL9*, *CKS1B* and *ILF2* deserve particular interest because each of these genes has been proposed as a candidate participant in myelomagenesis [34,36,46,63]. The identification of potential therapeutic targets, along with the proper risk stratification for patients with 1q21 amp, will not only provide the rationale for individualized therapies according to clinical risk, but also will also provide effective first-line treatments with a durable response for 1q21 MM patients who do not benefit from current therapies.

## Figures and Tables

**Table 1:** Biological function and implications of candidate genes in MM cells with 1q21 amp.

Candidate Genes	Chromosomal Location	Biological Function	Function of Overexpressed Gene in MM Cells with 1q21	References
*MCL-1*	1q21.2	Anti-apoptotic	Apoptosis resistance, disease progression, and shorter OS	[35,38]
*CSK1B*	1q21.3	Cell division	Regulation of the p27 proteasome degradation	[39,40]
*IL6R*	1q21.3	IL6 regulator	Increased IL6 sensitivity and hyper-activation of the STAT3 pathway	[34,41]
*ILF2*	1q21.3	mRNA stability, mRNA post-transcriptional regulation, and in mitotic control.	Promotes tolerance of genomic instability, enhancing MM cell survival and drug resistance	[36,42,43]
*BCL9*	1q21.2	T ranscriptional co-activator of the Wnt/β-catenin pathway	Promotes cell proliferation?	[44,45]
